# Designing a smallpox B-cell and T-cell multi-epitope subunit vaccine using a comprehensive immunoinformatics approach

**DOI:** 10.1128/spectrum.00465-24

**Published:** 2024-05-03

**Authors:** Changqing Yu, Qi Wu, Jiuqing Xin, Qiujuan Yu, Zhixin Ma, Mengzhou Xue, Qingyuan Xu, Chunfu Zheng

**Affiliations:** 1 State Key Laboratory for Animal Disease Control and Prevention, Harbin Veterinary Research Institute, Chinese Academy of Agricultural Sciences, Harbin, China; 2 Engineering Center of Agricultural Biosafety Assessment and Biotechnology, School of Advanced Agricultural Sciences, Yibin Vocational and Technical College, Yibin, China; 3 Department of Dermatology, The First People's Hospital of Mudanjiang, Mudanjiang, China; 4 Department of Cerebrovascular Diseases, The Second Affiliated Hospital of Zhengzhou University, Zhengzhou, China; 5 Department of Microbiology, Immunology and Infection Diseases, University of Calgary, Calgary, Canada; National Institutes of Health, Bethesda, Maryland, USA

**Keywords:** smallpox, variola virus, vaccine design, multi-epitopes vaccine

## Abstract

Smallpox is a highly contagious human disease caused by the variola virus. Although the disease was eliminated in 1979 due to its highly contagious nature and historical pathogenicity, with a mortality rate of up to 30%, this virus is an important candidate for biological weapons. Currently, vaccines are the critical measures to prevent this virus infection and spread. In this study, we designed a peptide vaccine using immunoinformatics tools, which have the potential to activate human immunity against variola virus infection efficiently. The design of peptides derives from vaccine-candidate proteins showing protective potential in vaccinia WR strains. Potential non-toxic and nonallergenic T-cell and B-cell binding and cytokine-inducing epitopes were then screened through a priority prediction using special linkers to connect B-cell epitopes and T-cell epitopes, and an appropriate adjuvant was added to the vaccine construction to enhance the immunogenicity of the peptide vaccine. The 3D structure display, docking, and free energy calculation analysis indicate that the binding affinity between the vaccine peptide and Toll-like receptor 3 is high, and the vaccine receptor complex is highly stable. Notably, the vaccine we designed is obtained from the protective protein of the vaccinia and combined with preventive measures to avoid side effects. This vaccine is highly likely to produce an effective and safe immune response against the variola virus infection in the body.

**IMPORTANCE:**

In this work, we designed a vaccine with a cluster of multiple T-cell/B-cell epitopes, which should be effective in inducing systematic immune responses against variola virus infection. Besides, this work also provides a reference in vaccine design for preventing monkeypox virus infection, which is currently prevalent.

## INTRODUCTION

Smallpox is an exceptionally lethal disease caused by variola virus, a member of the *Orthopoxvirus* genus of the *Poxviridae* family. It was described as “the most dreadful scourge of the human species.” Although the exact number of deaths is not recorded, it is estimated to amount to 400 million people in the 20th century alone (1). The first clinical descriptions of smallpox were recorded in China in the 4th century, and smallpox was observed in Europe and Asia before the 15th century. During colonialism, smallpox was imported by Europeans into the Americas, Southern Africa, and Australia between the 15th and 18th centuries (1). Smallpox is highly infectious and is thought to be transmitted both as an aerosol and via fomites. Usually, the virus enters the body by the inhalation of microdroplets shedding from the respiratory tract of infected persons (2–4). After 7–9 days post-infection, patients experience a prodromal phase with high fever, malaise, and headaches. As the disease progresses, the virus replicates in the mucosa and exhibits the most prominent rash characteristics of smallpox.

Smallpox has been announced to be eradicated in human history, and the vaccine played a critical role in successfully eliminating the variola virus infection (5). Due to the discovery that the symptoms of skin wound infection with variola virus are milder than those caused by respiratory infections, people are immunized through skin wound infection with the virus. This immunization method was first used in China and India, and till the 8th century, Europe and some European colonies also adopted this immunization strategy. In the late 18th century, it was discovered that the workers who milked the cows were exempted from this severe life-threatening disease and pock-marked skin characteristics (6). Based on this observation, Edward Jenner ushered in the era of vaccination with cowpox. Since then, multiple vaccine strains from animal sources have been used in different countries and regions (7). These vaccines have played crucial roles in preventing smallpox. However, their side effects cannot be ignored, especially for immunocompromised recipients, and a small portion of the population may experience neurological symptoms, such as encephalitis, after receiving the vaccine (8). Therefore, in order to reduce the side effects of immunization, researchers have engaged in a series of work. Currently, prophylactic vaccines for smallpox can be divided into three generations. The first generation represented by the Lister and Dryvax vaccines, though exhibited remarkable heterogeneity (9, 10), showed a significant reduction in side effects compared to previous ones. The second-generation vaccine is obtained by plaque purification based on the first one, such as the ACAM2000 vaccines approved in the United States in 2007. Subsequently, people developed modified vaccinia Ankara (MVA) (licensed in Germany) and Imvanex Vaccine (licensed in European Union, Iceland, Liechtenstein, Canada, and Norway), Jyneos Vaccine (licensed in the USA) and LC16m8 vaccine (licensed in Japan). As technology progressed, attempts have been made to develop third-generation vaccines, such as multi-variant smallpox DNA vaccine (11), protein-based smallpox vaccine (12, 13), and T-cell epitope vaccine (14). However, so far, the third-generation vaccines are still in the development stage.

**Fig 1:**
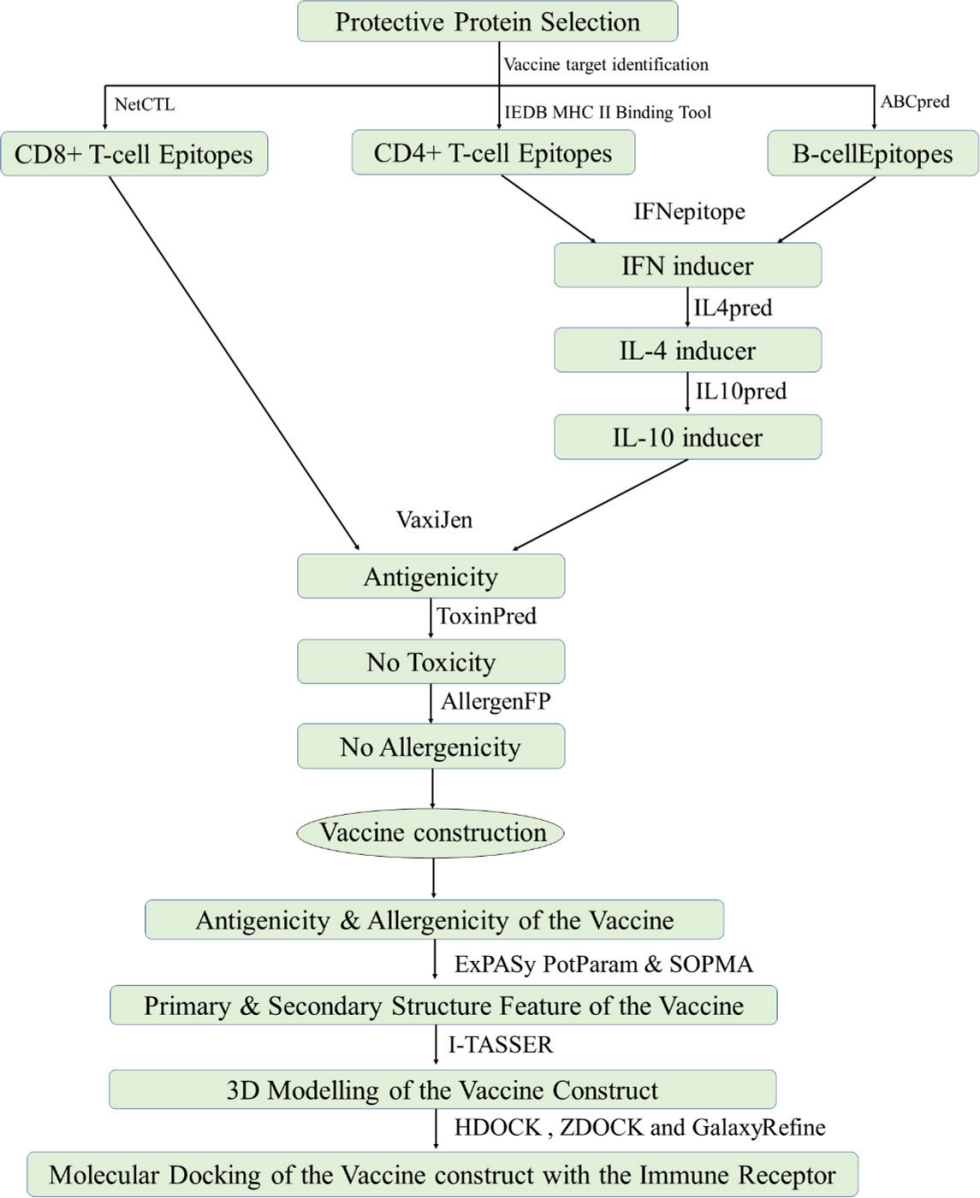
Systemic workflow of subunit vaccine designing using protective-related proteins.

Studies on vaccines, especially the novel ones that have been developed, are of great importance in preventing smallpox prevalence. In the design of new vaccines, both the effectiveness and a few side effects should be considered as a priority. In this study, proteins were screened according to their potency to induce neutralizing activities against the variola virus. Through a series of immunoinformatics analyses, we obtained a certain number of B-cell and T-cell epitopes with good humoral and cellular immune parameters. Furthermore, we screened epitopes that can induce immune-related cytokines without sensitization or cytotoxicity. These epitopes were connected with specific linkers to construct a multi-epitope vaccine. In addition, an adjuvant of β-defensin was added to the N-terminus of the above epitopes to enhance the immunogenicity. Finally, a series of analyses and evaluations were conducted on the constructed vaccines (Fig. 1).

## METHODS

### Smallpox immune-related protein

Protective proteins are crucial for the prevention of virus infection. To design a more protective vaccine, the proteins selected in this study were all smallpox neutralizing-immunity inducted proteins, which were confirmed in previous research, including L1 protein (GenBank: QTC35412.1) (2, 15), H3 protein (GenBank: QTC35394.1) (2, 16), B5R protein (GenBank: Q01227.1) (16–18), D8 protein (YP_232995.1) (2), and A27 protein (GenBank: QTC35423.1) (2).

### Identifying cytotoxic T lymphocyte epitopes

The peptides antigen that can cause cytotoxic T lymphocyte (CTL) cell responses are crucial in vaccine epitope design. To predict CTL epitopes in candidate proteins, this study used a NetCTL server (NetCTL 1.2 Services DTU Health Tech) (19) to predict CD8^+^ T-cell epitopes (20, 21), with weight on C terminal clearance set to 0.15, weight on TAP transport efficiency set to 0.05, threshold for epitope identification set to 0.75, and predicted CTL epitopes were restricted to 12 MHC-I subtypes.

### Identifying helper T lymphocyte epitopes

T helper cell 15-mer epitopes for human MHC-II alleles (a reference panel of 27 alleles) were predicted for all the selected proteins using Immune Epitope Database (IEDB) (IEDB.org) with NN-align 2.3 method (22, 23). The IC_50_ values of 50, 500, and 5,000 nM indicated high, intermediate, and low affinity of epitopes, respectively (24). Among the predicted helper T lymphocyte (HTL) epitopes of each protein with IC_50_, less than 50 and affinity ranking in the top 50 were considered for constructing peptide vaccines (25).

### B-cell epitopes prediction

The ABCpreds server was used to predict 16-mer linear B-lymphocyte (LBL) epitopes with a threshold of 0.51, and only non-overlapping epitopes were predicted.

### Antigenicity, allergenicity, and toxicity assessment of identified epitopes

The antigenic potential of T-cell and B-cell epitopes was analyzed by VaxiJen v2.0 with a threshold of 0.4 (26). The T-cell and B-cell epitopes were further predicted in terms of toxicity and allergenicity with online software ToxinPred (27) and AllergenFP v.1.0 (28). Furthermore, the ability to induce the secretion of cytokines, including interferon-gamma (IFN-γ), interleukin-4, and interleukin-10, was predicted by IFNepitope (29), IL4Pred (30), and IL10 (31), respectively.

### Construction of the multi-epitope polypeptide

To construct a multi-epitope vaccine, the predicted epitopes that demonstrated high antigenic potential but without allergic or toxic features were obtained. These HTL and LBL epitopes were further analyzed for their ability to induce IFN-gamma, IL-4, and IL-10 production. Then, the selected epitopes were fused with different linkers. The CTL and LBL epitopes were linked with linker “KK,” HTL epitopes with “GPGPG,” and β-defensin was fused with the N-terminal tail of the vaccine through “EAAAK” to increase the immunogenic capacity.

### Physicochemical properties and secondary structure prediction

The physicochemical properties and secondary structural features are highly associated with the functionality of proteins. To compute various physicochemical properties and secondary structural features of the multi-epitope vaccine, the ExPASy ProtParam and PSIPRED were employed with default parameters.

### Immunogenic, allergenic, and physiochemical evaluation of vaccine construct

The antigenicity of the multi-epitope vaccine was analyzed by Vaxijen v2.0 online software with a threshold value of 0.4. The allergenicity of this vaccine was analyzed with AllerTOP v.2.0 and AllerTop v2.0 servers (32, 33). The physical chemistry properties of the vaccine were evaluated by ProtParam, including amino acid composition, molecular weight, theoretical isoelectric point, grand average of hydropathicity (GRAVY), aliphatic index and instability index, and *in vitro* and *in vivo* half-life.

### Vaccine polypeptide structure modeling, refinement, and validation

The second structure of the multi-epitope vaccine was analyzed using the SOPMA server (34). The I-TASSER server (35, 36) and the GalaxyWEB server (37) were employed to model and refine the vaccine’s 3D structure model. The predicted model structures were relaxed by packing and molecular dynamics (MD) simulation with the GalaxyWEB server (37). Next, the SAVES 6.0 server (38–42), which contains five evaluation algorithms, was employed to validate the refined 3D model for the final structure selection.

### Molecular docking of vaccine with immune receptor

Immune receptors play an important role in recognizing the antigenic molecules for inducing an appropriate immune response (21). To virus infection, the Toll-like receptors-3 is a crucial receptor that enhances the antiviral response by its ability to sense double-stranded RNA intermediates. For analyzing the binding pattern of the multi-epitope vaccine with TLR-3(PDB ID:2A0Z), molecular docking was performed by HawkDock (43), TongDock (44), HDOCK (45), and ZDOCK (46). The first docking complex ranked by each software is selected, and HawkDock is used to calculate the free energy of these docking products. The docking compound with the lowest free energy is taken as the final docking product. The hydrogen bonding formation was analyzed using the structure analysis model of ChrimeraX (47).

## RESULTS

### CTL epitope prediction

The MHC-I epitopes of candidate proteins L1, H3, B5, D8, and A27 were predicted by NetCTL 1.2 server. In the prediction process, we analyze all 12 supertypes. Then, the VaxiJen 2.0 server, ToxinPred, and AllergenFP were used to identify the antigenicity, toxicity, and allergy of CTL epitopes. All epitopes with antigenicity greater than 0.9 and not allergenic were selected as candidate epitopes, and 29 epitopes were finally obtained (Table 1).

**TABLE 1:** CTL epitopes for multi-epitope vaccine construction

Protein	Peptide sequence	Supertype	Antigen	Toxin prediction	Allergenicity prediction
			Prediction	(SVM)	
A27	QRLTNLEKK	B27	1.1973	−1.18	NO
B5	FSIGGVIHL	A26/B39/	1.2283	−1.15	NO
		B58/B62			
B5	CPNAECQPL	B7	1.0961	−0.09	NO
B5	KKMCTVSDY	B27	0.9203	−0.23	NO
B5	GKWNPVLPI	B27	0.9397	−0.9	NO
B5	YEVNSTMTL	B39/B44	0.9371	−0.87	NO
B5	IDGKWNPVL	B39	1.4244	−0.62	NO
B5	FNDKQKVTF	B39	1.4542	−0.74	NO
B5	LSCKSGFTL	B58	0.9732	−1.08	NO
D8	LSDLRETCF	A1	1.9684	−0.81	NO
D8	VLDHKNVYF	A1	1.7004	−1.55	NO
D8	RLKPLDIHY	A1/A3/B58/B62	2.9399	−1.41	NO
D8	AILFFMSRR	A3	1.0547	−0.79	NO
D8	NLVHWNKKK	A3	1.232	−0.77	NO
D8	EINLVHWNK	A3	1.3749	−0.62	NO
D8	DLRETCFSY	A26/B62	2.5549	−0.95	NO
D8	VRINFKGGY	B27	2.2055	−1.04	NO
D8	VLSSLHIYW	B58	1.2297	−1.48	NO
D8	ISNARLKPL	B62	1.6231	−1.18	NO
H3	ELENKKVEY	A1/A26	1.6116	−0.65	NO
H3	MLIFNVKSK	A3	1.8488	−1.4	NO
H3	MHDKKIDIL	B39	1.5062	−1.1	NO
H3	YKDYAFIQW	B39	1.5672	−0.85	NO
L1	FYMIVIGVI	A24	1.1164	−0.93	NO
L1	ENVHWTTYM	A26	1.1287	−0.71	NO
L1	CDIEIGNFY	A26	1.1561	−1.27	NO
L1	KLKIQNVII	B8	0.9932	−0.99	NO
L1	KENVHWTTY	B44/B62	1.1133	−0.57	NO
L1	LANKENVHW	B58	1.7164	−0.67	NO

### HTL epitope prediction

T helper cell 15-mer epitopes for human MHC-II alleles were predicted for all the selected proteins of the vaccinia virus using the Immune Epitope Database (IEDB) (IEDB.org) with NN-align 2.3 method (22, 23). All 27 alleles were selected in the prediction, and the allele population coverage reached 99%. Among the predicted HTL epitopes of each protein, those with IC_50_ less than 50 and affinity ranking in the top 50 were considered to be used to construct peptide vaccines (25). Based on these results, five epitopes were picked up via further analyzing their antigenicity, toxicity, allergenicity, IFN induction ability, IL-4 induction ability, and IL-10 induction ability, among which three were duplicated and thus removed. The remaining two HTL epitopes were selected for vaccine construction (Table 2).

**TABLE 2:** HTL epitopes for multi-epitope vaccine construction

Protein	Peptide sequence	MHCII HLA alleles	Antigen prediction	Toxin prediction (SVM)	Allergenicity prediction	IFN inducer (Score)	IL-4 inducer (Score)	IL-10 inducer (Score)
D8	ETKKAISNARLKPLD	HLA-DRB5*01:01	1.2689	−1.8	NO	0.33	0.73	0.71
L1	KIKLILANKENVHWT	HLA-DRB1*13:02 HLA-DRB1*01:01	1.3608	−1.08	NO	0.49	0.47	0.36

### LBL epitopes prediction

The ABCpred server was used to predict the LBL of five proteins (L1, H3, B5R, D8, and A27) with a length of 16mer. When the threshold was set to 0.51, and duplicate epitopes were filtered out, 127 epitopes were obtained, including 21 from L1, 31 from H3, 32 from B5R, 32 from D8, and 11 from A27. After a screening of antigenicity, toxicity, allergenicity, IFN induction ability, IL-4 induction ability, and IL-10 induction ability, five epitopes were finally screened to construct a peptide vaccine (Table 3). Among them, one epitope was screened for L1 protein, three epitopes were screened for H3 protein, and one epitope was screened for A27 protein, while epitopes satisfying the above restrictions were nonexistent in B5 and D8 proteins.

**TABLE 3:** LBL epitopes for multi-epitope vaccine construction

Protein	Sequence	Score		Toxin prediction (SVM)	AllergenFP	IFN inducer (Score)	IL-4 inducer (SVM Score)	IL-10 inducer (Score)
L1	SERISSKLEQEANASA	0.9	1.3723	−1.41	NO	0.16	0.83	0.40
H3	FEIARIENEMKINRQI	0.69	0.6155	−1.08	NO	0.24	1.37	0.69
H3	GGLSSGFYFEIARIEN	0.73	0.6475	−1.23	NO	0.17	1.45	0.47
H3	DPRLVAEHRFENMKPN	0.77	1.0702	−0.95	NO	0.24	0.26	0.45
A27	FRLENHAETLRAAMIS	0.66	0.9229	−1.18	NO	0.35	0.43	1.20

### Multi-epitope vaccine polypeptide construction

**Fig 2:**
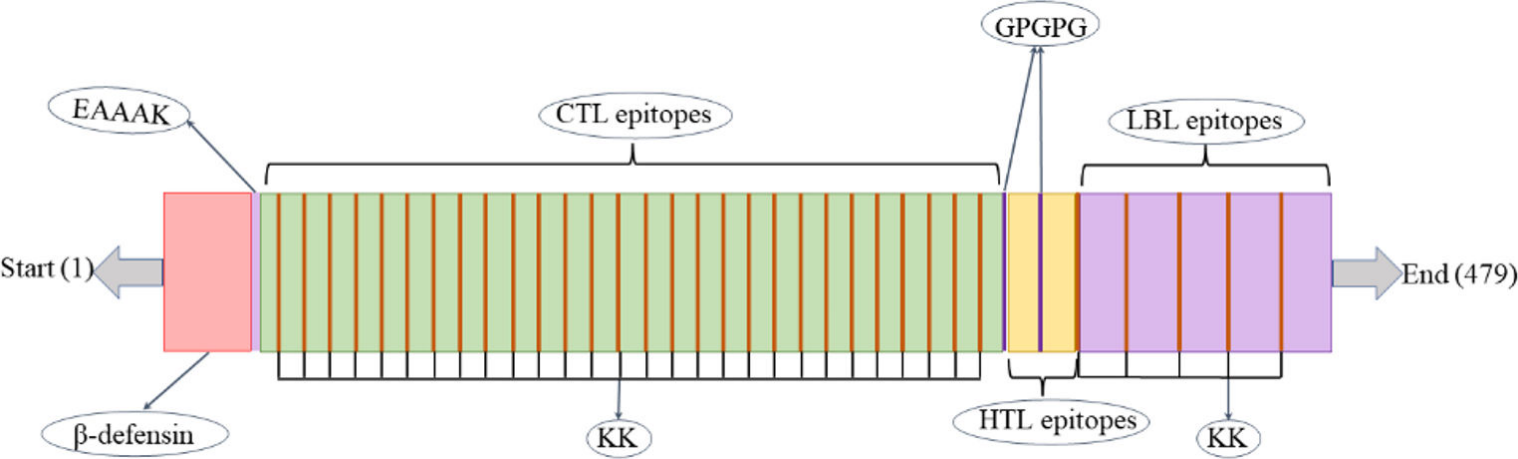
Schematic profile of the multi-epitope vaccine construct of length 479 residues. β-defensin was added at the N-terminal tail of the vaccine using an EAAAK linker, followed by 29 CTL, 2 HTL, and 5 LBL. The epitopes of CTL, HTL, and LBL were linked by KK, GPGPG, and KK, respectively.

In the construction of a peptide vaccine, the predicted CTL epitopes with good antigenicity, non-toxic, and allergenicity were selected for peptide vaccine construction. For HTL epitopes and LBL epitopes, besides the selection criteria in CTL epitopes, cytokine-induced indicators (including IFN, IL-4, and IL-10) are also added, which has been expected to achieve better immune effects. Twenty-nine CTL epitopes and 2 HTL epitopes were connected through KK and GPGPG linker, respectively. Then, five LBL epitopes linked by KK were constructed to the C-terminal of the vaccine. To further improve the immune effect of the vaccine, the first CTL epitope at the N-terminal of the polypeptide vaccine was introduced with an EAAAK linker β-defensin sequence (GIINTLQKYYCRVRGGRCAVLSCLPKEEQIGKCSTRGRKCCRRKK) (Fig. 2).

### Immunogenic, allergenic, and physiochemical evaluation

The antigenicity of the constructed vaccine was analyzed by a VaxiJen 2.0 server with a threshold of 0.4 as the parameter, and the score was 0.5880, which screened the vaccine that had good antigenicity. The AlgPred server was employed to predict the allergenicity of the vaccine by mapping IgE allergens and PID. The results indicated that the construction does not contain empirically proven IgE allergens. The allergenicity of the vaccine construct was cross-checked through AllerTOP v2.0 and indicated a non-allergic nature. These results provided strong evidence for the non-allergic feature of this vaccine construct.

### Physicochemical properties and secondary structure analysis

Through the ExPASy ProtParam Tool, various physical and chemical properties of multi-epitopes were analyzed. The molecular weight of the construct is 58.67 kDa and thus meets the basic conditions of antigen. The protein isoelectric (PI) point provides a reference for the pH value of the protein purification buffer system. The PI is about 10.19, and its buffer solution should be slightly alkaline. In addition, the number of negative and positive amino acid residues is 43 and 134, respectively. The extinction coefficient was 80,330 M^−1^ cm^−1^ at 280 nm measured in water, assuming all cysteine residues are reduced. The estimated half-life is 30 h in mammalian reticulocytes (*in vitro*), 20 h in yeast (*in vivo*), and 10 h in *Escherichia coli* (*in vivo*). The instability index is computed to be 33.87, indicating that the vaccine construct is classified as a stable protein. The aliphatic index and GRAVY are 77.08 and −0.831, respectively, illustrating that the protein has good thermal stability and water solubility. The secondary structural features of the construct were assessed via SOPMA using the amino acid sequence of length 497 of the construct. SOPMA prediction results show that alpha helixes account for 33.20%, beta strands account for 25.75%, and random coils account for 29.18% in the whole sequence. These results indicate that our construct can form different intra- and inter-chain structures and different types of secondary structures.

### Tertiary structure prediction

**Fig 3:**
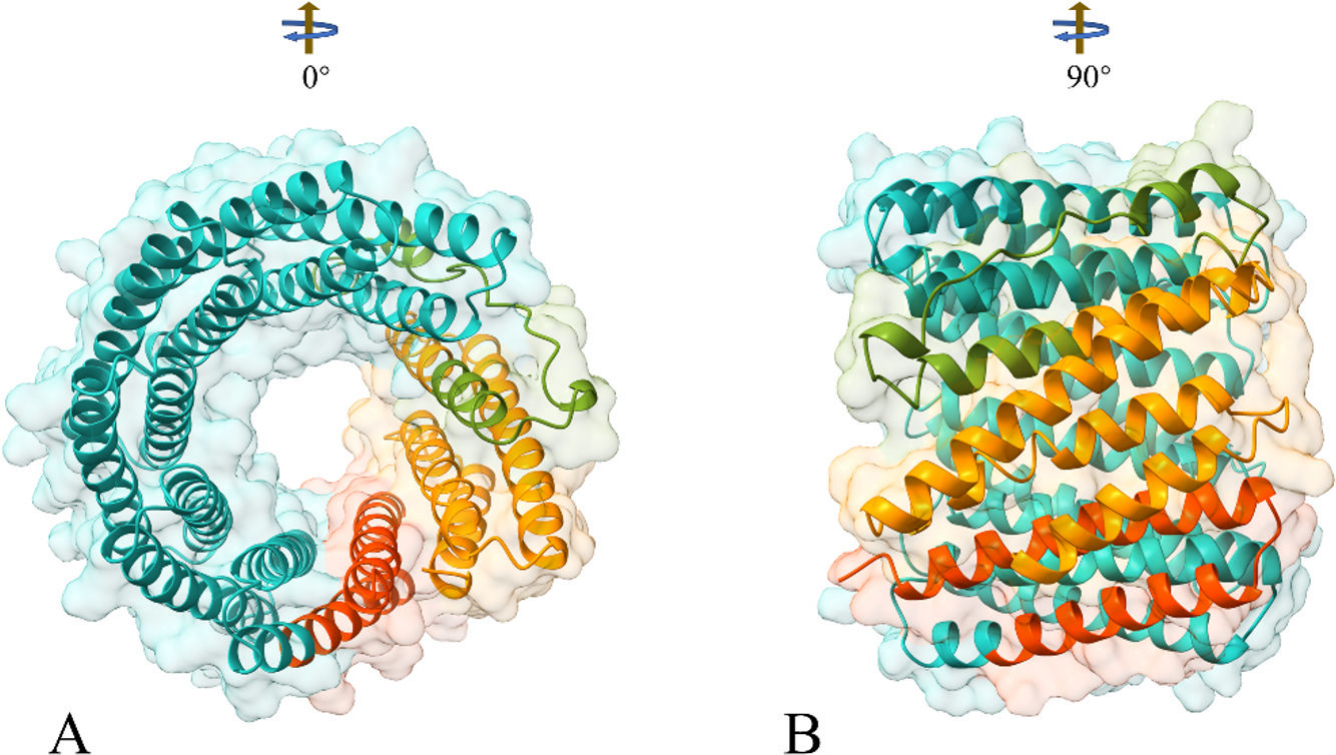
The final 3D model of the multi-epitope vaccine was obtained after I-TASSER prediction and refinement with the GalaxyWEB server. The final composite forms a ring-like structure, with (A) and (B) showing different perspectives of the model. In the figure, β-defensin is marked in orange-red, CTL is marked in light sea green, HTL is marked in olive drab, and LBL is marked in dark orange.

**Fig 4:**
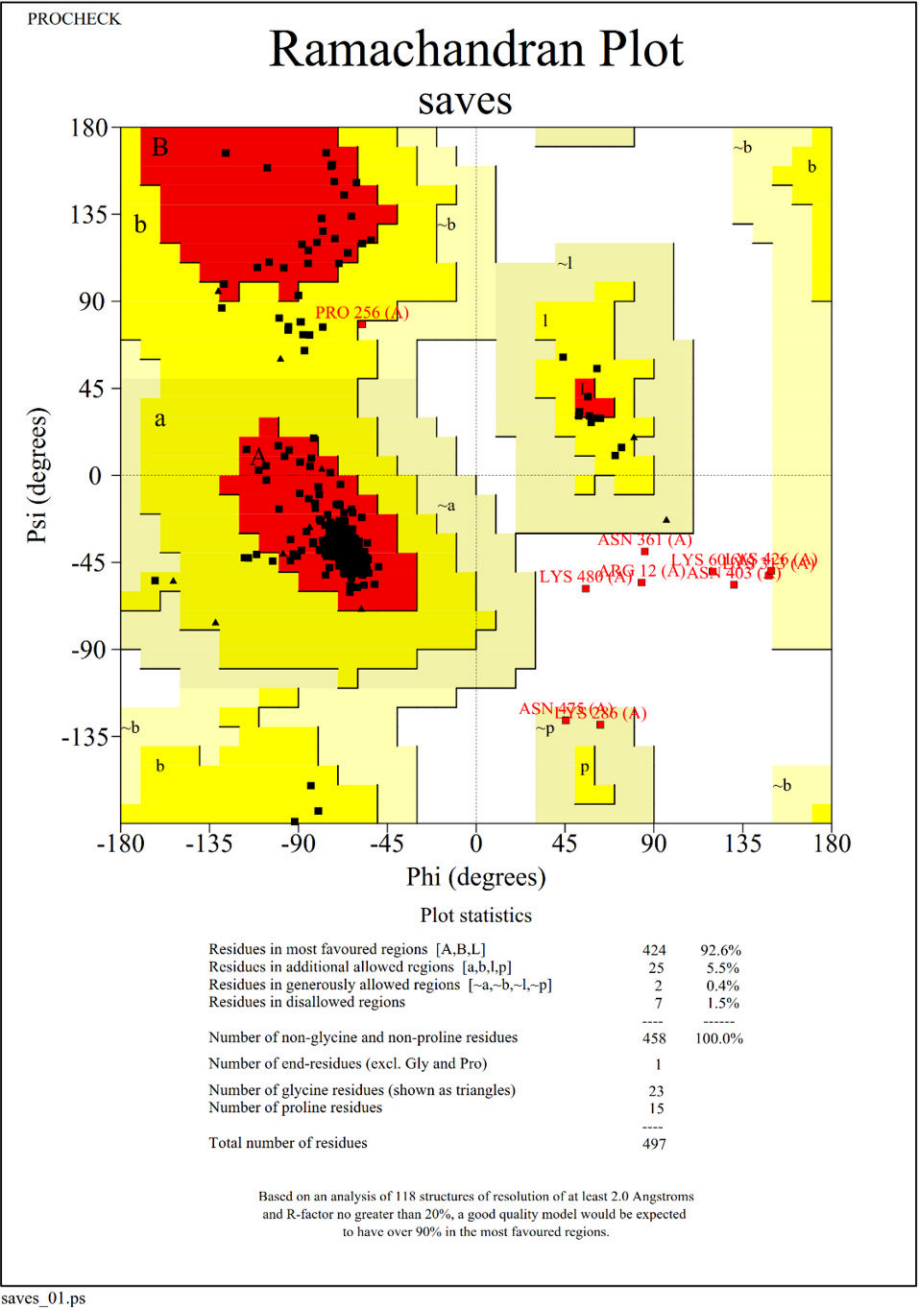
Validation of the predicted 3D model of the final vaccine construct. The refined 3D structure of the multi-epitope vaccine was validated by the generation of the Ramachandran plot, where 92.6% residues were found to lie in the favored region, whereas 5.5% and 0.4% residues were there in additional and generously allowed regions. Only 1.5% were in the outlier region.

Since there is no ideal template for homologous modeling, the I-TASSER server was used to predict the 3D structure of the constructed vaccine. The C-scores of the first five models predicted by the I-TASSER server are -0.209,–1.53, -0.99,–2.57, and −4.18, respectively. In the I-TASSER 3D structure prediction, the C-score is usually in the range of −5 to 2. The higher the score is consistent, the higher the reliability of the model, and vice versa. The GalaxyRefine server was used to optimize the model. The GalaxyRefine provided five optimization models based on different qualities for each basic model. Then SAVES server was used to evaluate all models, and the third model optimized by Galaxy Reinfe based on I-TASSER predicted model3 was selected as the optimal vaccine 3D model (Fig. 3). The Ramachandran plot analysis validated the final construct model with 92.6%, 5.5%, 0.4%, and 1.5% residues in most Rama-favored regions, additional allowed regions, generously allowed regions, and disallowed regions, respectively (Fig. 4). The overall quality factor was 91.79, produced by ERRAT.

### Molecular docking of final vaccine construct with immune receptor TLR3

**Fig 5:**
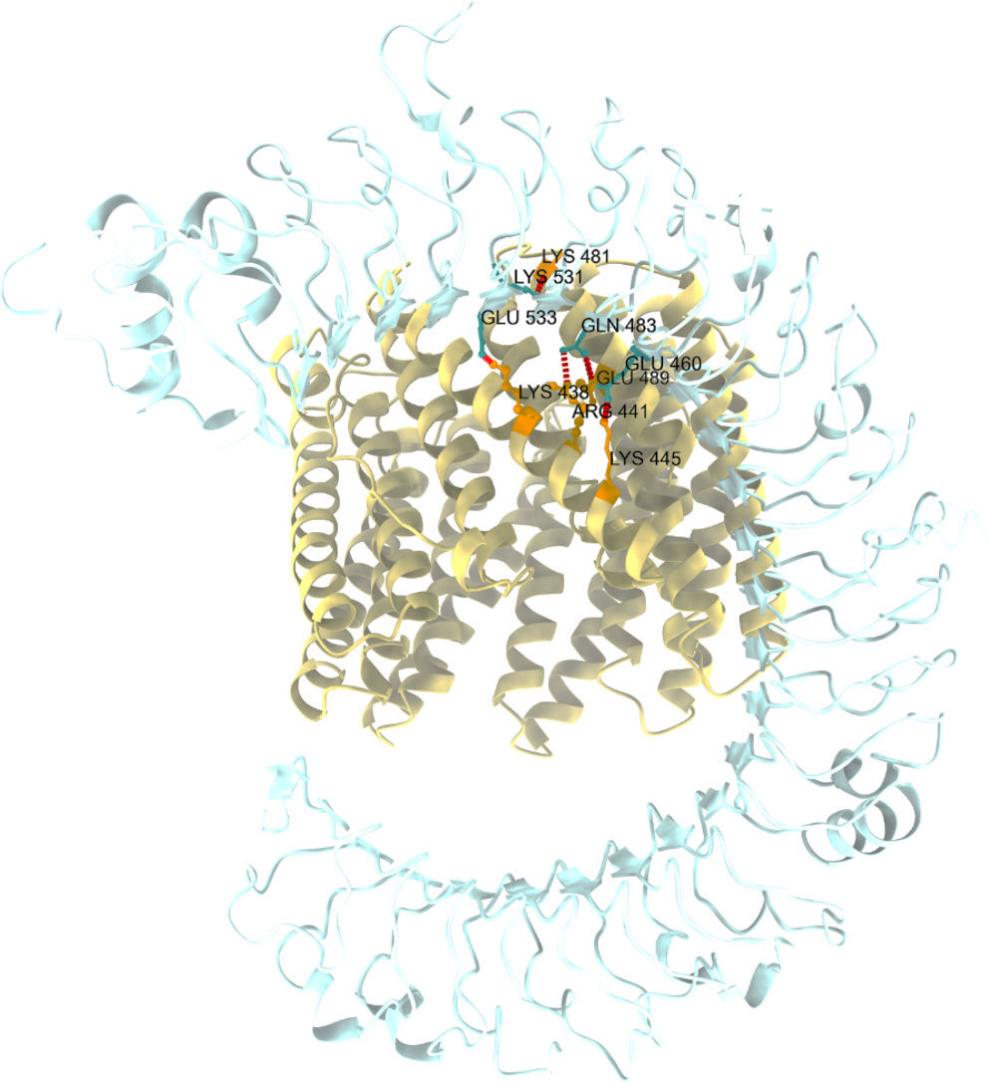
A docked complex of TLR-3 (PDB ID: 2A0Z) with the multi-epitope vaccine. The receptor (TLR-3) has been depicted in light cyan, whereas the khaki color depicts the multi-epitope vaccine as a ligand in the docked complex obtained from molecular docking. The hydrogen bond between the vaccine and TLR3 is displayed in red. The residues involved in hydrogen bonding in vaccines are colored orange, while the amino acids involved in hydrogen bonding in TLRS are shown light sea green.

The docking of TLR3 with the optimal 3D model of the vaccine was performed with four software: HawkDock (43), TongDock (44), HDOCK (45), and ZDOCK (46). To screen the best docking model, the first docking model that come from each docking software was further analyzed by the HawkDock software. The docking compound with the lowest energy should be chosen as the final model. The free energies of HDOCK, ZDOCK, HawkDock, and TongDock docking products were −23.51, −57.1, −66.71, and −81.53 kcal/mol, respectively. The docking products of TongDcok were selected as the final docking complex for the lowest free energy (Fig. 5). The hydrogen bonding of the protein docking complexes was analyzed with the structure analysis module of the software Chimera X. Analysis of residues at the protein-protein interface showed that five hydrogen bonds were formed between the vaccine residues of LYS438, ARG441, LYS445, GLU489, LYS481, and the TLR3 residues of GLU533, GLN483, GLU460, and LYS531.

## DISCUSSION

Smallpox is an exceptionally lethal disease caused by the variola virus, and the mortality of this disease is generally no more than 30% (2). Given the high mortality rate of smallpox infection and the endemic nature of the disease, it may have caused more human deaths during the past two millennia than any other single disease. After vaccination campaigns throughout the 19th and 20th centuries, the WHO declared the eradication of smallpox in 1979 (48). At that time, the vaccinia was obtained from calves inoculated with live viruses, whereas the manufacturing process was not in good compliance with the standards acceptable nowadays (49). This type of vaccine contains live viruses that may lead to multiple side effects, such as fever, mild rash, eczema vaccinatum, progressive vaccinia, and encephalitis (50–52). Therefore, tremendous efforts have been made to develop novel vaccines for improving safety. Various vaccines have been reported, not only traditional vaccines [such as ACAM2000 (53–55), Elstree-BN (56), CCSV (57), Imvamune (58), NYVAC (59), and LC16m8 (60)] but also next-generation vaccine [including DNA vaccines (11), protein subunit vaccines (12, 13), and T-cell epitope vaccines (14)].

In this study, we selected L1, H3, B5, D8, and A27 proteins from the vaccinia WR strain for epitope analysis, all of which are closely related to the immune protection of smallpox. Among them, L1, H3, and A27 proteins are neutralizing antibody targets, B5 protein is related to serum neutralizing activity, and D8 protein has a synergistic effect with L1 and a protective effect in mouse models (2, 15–18). More interestingly, these proteins are highly conserved among the vaccinia virus, variola virus, and monkeypox virus, suggesting that these proteins may induce cross-protection against these viruses. In the development of a new generation of vaccines, besides focusing on immune protection indicators, the side effects of vaccines should also be considered. This study obtained B-cell and T-cell epitopes through bioinformatics analysis of these highly immunoprotective proteins and then removed the epitopes that may cause allergic reactions or toxicity, which not only reduces the length of the existing protein, making expression easier but, more importantly, can minimize the side effects of the vaccine, which is also the main problem that vaccinia immunization has encountered before.

When analyzing the structure of peptide vaccines, the results of SOPMA were different from those of I-TASSER, which should be due to algorithmic differences. Epitope-based peptide vaccines may have weak immune responses when used alone (61). Therefore, when designing epitope peptide vaccines, appropriate adjuvants are often added to enhance the immune response (62–64). β-defensins have various immune activities, and in addition to their direct killing effect on pathogenic microorganisms, they can also serve as chemokines to enhance and alter adaptive immune responses (65). Vaccines containing defensins as adjuvants have been shown to activate the primary innate antiviral immune response *in vivo* and *in vitro* and mediate other immune regulatory activities against various viruses, including coronaviruses (66, 67). The use of appropriate adjuvants has also been shown to help induce persistent IFN-γ reactions (68). Therefore, in order to improve the efficacy of epitope peptide vaccines, the β-defensin was fused with epitope peptides in this study. In addition, we evaluated the binding of the constructed vaccine model to the immune receptor TLR-3 through molecular docking and free energy calculation. Triggering TLR-3 may help induce TLR signaling networks, which activate specific immune pathways targeting the evolution of viral pathogens.

## Data Availability

The authors confirm that the data supporting this study are available within the article.
